# Feasibility of respiratory synchronization for laser lithotripsy using a robotic retrograde intrarenal surgery system Zamenix™ in an in-vitro model

**DOI:** 10.1186/s40001-025-02395-9

**Published:** 2025-04-04

**Authors:** Joonhwan Kim, Chinnakhet Ketsuwan, Kyu-Seob Song, Jae-chul Kim, Joonyeong Kim, Hyeji Park, Dong-Soo Kwon, Joo Yong Lee, Sung Yong Cho

**Affiliations:** 1 ROEN Surgical, Inc., Daejeon, Korea; 2 Division of Urology, Department of Surgery, Faculty of Medicine Ramathibodi Hospital, Mahidol University, Bangkok, Thailand; 3 Department of Urology, Seoul National University Hospital, Seoul National University College of Medicine, 101, Daehak-Ro, Jongno-Gu, Seoul, 03080 Republic of Korea; 4 Department of Mechanical Engineering, Korea Advanced Institute of Science and Technology (KAIST), Daejeon, Korea; 5 Department of Urology, Severance Hospital, Urological Science Institute, Yonsei University College of Medicine, Seoul, Korea

**Keywords:** Robotics, Respiration, Urologic surgical procedures, Ureteroscopy, Urolithiasis

## Abstract

**Objectives:**

This study aims to investigate the improvement of stone fragmentation efficiency and safety in robotic-assisted retrograde intrarenal surgery (RIRS) that implements the respiratory motion synchronization using an in vitro model.

**Materials and methods:**

Laser lithotripsy was performed in three groups: manual procedure (group M), robotic procedures without respiratory synchronization (group RNR), and robotic procedures with respiratory synchronization (group RR). The study assessed fragmentation time, laser time, number of mucosal contacts, and total energy used. Two surgeons having different experience of conventional RIRS (> 2500 and < 500) were participated.

**Results:**

In overall results of the two surgeons, the fragmentation time significantly decreased to 74.8% in group RNR (*P* = 0.012) and 65.0% in group RR (*P* = 0.001), compared to group M. The laser time was significantly shorter in group RR compared to the group M (*P* = 0.003). The number of mucosal contacts was significantly reduced to 37.4% in group RNR (*P* = 0.048) and it was 34.0% in group RR, compared to group M. The total energy significantly decreased in group RR compared to group M (*P* = 0.011). There were no significant differences between group RR and RNR across all outcomes in the overall results of the two surgeons. For less experienced surgeon, the fragmentation time was significantly shorter in group RR compared to group RNR (*P* = 0.013).

**Conclusions:**

Robotic-assisted RIRS resulted in reduced fragmentation time, laser time, mucosal contacts, and total energy compared to manual RIRS during laser lithotripsy. The incorporation of respiratory synchronization in robotic-assisted RIRS reduced laser time compared to manual RIRS and shortened the fragmentation time compared to the robotic-assisted RIRS without respiratory synchronization, particularly for less experienced surgeon. These initial results demonstrated the feasibility of robotic-assisted RIRS with respiratory synchronization, highlighting its potential to improve procedural efficiency and safety.

**Supplementary Information:**

The online version contains supplementary material available at 10.1186/s40001-025-02395-9.

## Introduction

Retrograde intrarenal surgery (RIRS) utilizing a flexible ureteroscope presents a favorable option for the management of renal stone disease, offering an effective alternative to conventional percutaneous nephrolithotomy (PCNL) or shockwave lithotripsy [1, 2]. Compared to PCNL, RIRS is associated with lower morbidity, particularly in terms of blood loss and adjacent organ injuries, and it can achieve higher stone-free rates than shockwave lithotripsy [3]. Currently, RIRS is the standard treatment for renal calculi measuring 2 cm or smaller, with shockwave lithotripsy also being performed in some cases [4].

RIRS is typically performed under general anesthesia with mechanical ventilation assistance. However, regardless of the chosen anesthesia technique, one of the significant challenges encountered during RIRS is the displacement of stones within the operative field, which caused by kidney movement due to diaphragm and chest respiratory motions induced by mechanical ventilation. This respiration-induced kidney movement complicates the procedure, making it difficult to maintain a stable and consistent distance between the laser fiber tip, the stone, and the surrounding mucosa during the laser lithotripsy [5]. These challenges can prolong surgical time, and inaccurate targeting resulting from kidney motion may cause urothelial damage and subsequent complications.

To manage moving organs and stones during RIRS, techniques must be employed to avoid blood vessels and other structures while effectively controlling organ motion. Such measures are crucial for enhancing treatment effectiveness and minimizing damage to surrounding tissues. Previous studies have shown that reducing respiration-induced kidney movement can enhance stone fragmentation rates during RIRS. For instance, modifying respiratory rate and tidal volume during RIRS has been shown to significantly decrease renal mobility and improve fragmentation efficiency [5]. Although surgeons can attempt to control the ureteroscope to track kidney motion and maintain a consistent distance between the laser fiber tip, the stone, and the surrounding mucosa, this task is hindered by operator hand tremors and fatigue.

Robotic ureteroscopy systems has demonstrated potential for advancing RIRS techniques. Recent developments in robotic systems [6–8] have yielded comparable surgical outcomes with added benefits, such as improved ergonomics, tremor elimination, and reduced radiation exposure. However, while these robotic systems have primarily focused on the feasibility and safety validation for RIRS, their application in addressing respiration-induced kidney motion remains unexplored.

This study aims to investigate the feasibility of robotic-assisted laser lithotripsy with respiratory synchronization. Given the importance of mitigating respiration-induced movement in RIRS, the study evaluates the efficiency and safety of stone fragmentation, laying the groundwork for enhancing surgical outcomes in robotic-assisted RIRS.

## Materials and methods

### Robotic retrograde intrarenal surgery system, Zamenix™

Zamenix*™* (Renamed from easyUretero, ROEN Surgical, Inc. Daejeon, South Korea) is a teleoperated robotic system for RIRS. The system accommodates a commercial flexible ureteroscope, a commercial laser fiber, and a robotic stone basket, all of which are mounted to the robotic arm. A single seated operator can control the flexible ureteroscope, stone basket, and laser fiber through a console handles. Detailed features of the system have been previously described in publications [8, 9]. During the laser lithotripsy, the surgeon can control the ureteroscope (insertion/withdrawal, deflection, rotation) and laser fiber (insertion/withdrawal) via the console handles. Because the ureteroscope is mounted on the robotic arm, it remains stably positioned, unaffected by the operators’ hand tremor or fatigue. To accurately target the stone during the lithotripsy, the operator can control the position and orientation of the ureteroscope using the console handle. In addition, the console handle features a scroll wheel, enabling precise control of the laser fiber tip’s position. This allows the operator to maintain a consistent and safe distance between the laser fiber tip, the stone, and the surrounding mucosa during laser lithotripsy.

### In-vitro setup with kidney simulator

**Fig. 1:**
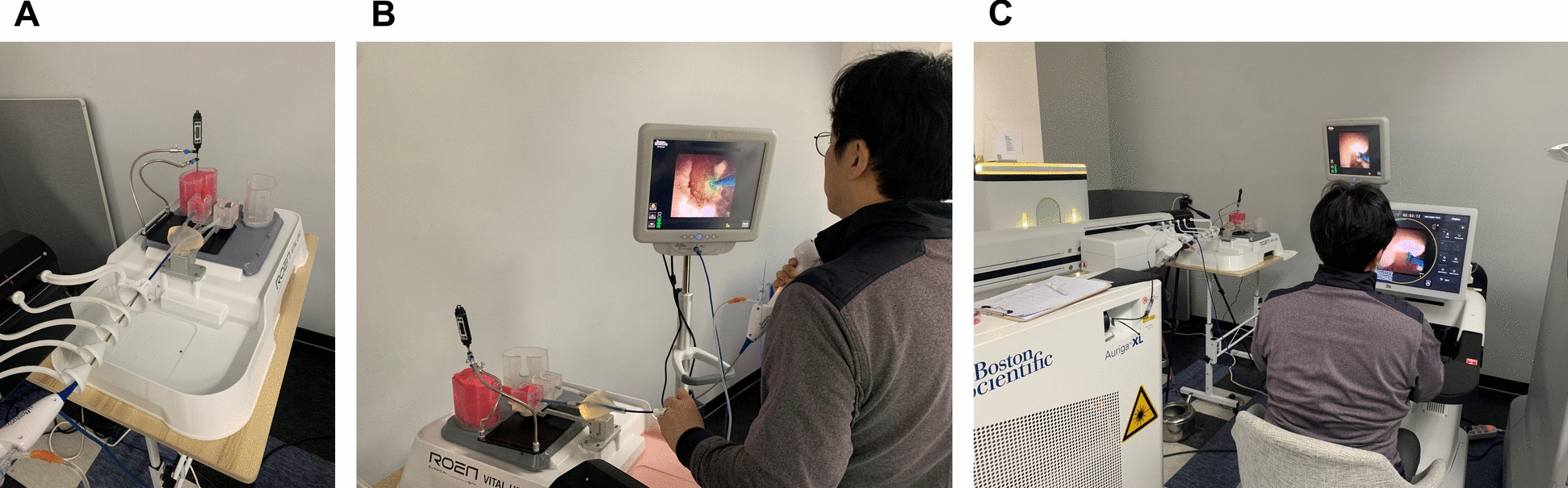
Experimental setup. **a** In-vitro kidney simulator. **b** Manual procedure (group M). **c** Robotic procedures with and without respiratory synchronization (group RNR and RR)

A novel simulator (ROEN Surgical, Daejeon, South Korea) designed to replicate respiration-induced kidney motion was utilized in this study. Respiration was simulated at a rate of 12 breaths per minute, with each cycle lasting 5 s. The amplitude of the kidney motion was randomly set to 10, 15, or 20 mm. The kidney model includes the renal pelvis, upper, mid, and lower poles. An artificial phantom stone, mimicking a 100% calcium oxalate stone, was fabricated with dimension of 5 × 5 × 5 mm. A single stone was positioned in the upper pole of the right kidney for each session. A thermometer was placed at the renal pelvis to measure the intrarenal temperature. Figure 1a illustrates the experimental setup with the in-vitro kidney simulator.

### Subjects and participants

Two surgeons participated in the experiment: a more experienced surgeon with over 2500 cases of RIRS (Cho SY, surgeon A) and a less experienced surgeon with fewer than 500 cases of RIRS (Ketsuwan C, surgeon B). Both surgeons performed 1–2 robotic-assisted laser lithotripsy trials prior to the experiments to familiarize themselves with the control interface.

### Surgical procedure

A flexible ureteroscope LithoVue (Boston Scientific, Marlborough, MA, USA) was inserted into the kidney simulator and advanced to the upper pole, where the stone was located. Stones were fragmented using the AURIGA XL laser machine (Boston Scientific, Marlborough, MA, USA) with laser settings of 0.8 J and 18 Hz (14.4 W). A 270 μm laser fiber was utilized for the procedure. During lithotripsy, participants were instructed to employ the pop dusting technique to achieve stone-free status. The participants were directed to maintain continuous laser emission for three respiratory cycles (15 s) followed by a pause for one respiratory cycle (5 s), repeating this pattern throughout the procedure to manage the intrarenal temperature elevation. The intrarenal temperature was monitored, and if it exceeded 48 °C, the participants were instructed to cease the laser firing until the temperature dropped below 48 °C. The irrigation pressure was set at 50 cm H_2_O (equivalent to 36.8 mmHg). The experiment consisted of three groups: (1) manual procedure (group M); (2) robotic procedure without respiratory synchronization (group RNR); and (3) robotic procedure with respiratory synchronization (group RR). In group M, the participants performed routine laser lithotripsy without the laser fiber control for the respiratory synchronization. In group RNR, the participants used the robotic system but did not adjust the laser fiber for the respiratory movement. In group RR, the participants used the robotic system and adjusted laser fiber to account for respiratory movements. Respiratory synchronization in the study was defined as a technique involving the insertion and withdrawal of the laser fiber in response to respiration-induced kidney motion, ensuring a consistent and stable distance between the laser fiber tip and the stone during the fragmentation. Operators measured the distance change between the laser fiber tip and the stone over a few respiration cycles, then manually controlled the position of the laser fiber tip in synchronization with respiration motion using the scroll wheel on the console handle. Each participant performed five sessions in in group M and seven sessions in groups RNR and RR. The sequence of group allocation was randomized. Figure 1b illustrates the manual procedure (group M), and Fig. 1c depicts the robotic procedures with and without respiratory synchronization (groups RNR and RR, respectively).

### Experimental outcomes

Fragmentation time (seconds), laser time (seconds), number of mucosal contacts, and total energy (Wh) were measured during the procedure. Fragmentation time was measured using a stopwatch and defined as the duration from the start of laser emission to the achievement of stone-free status (< 1 mm). Stone-free status was determined when no residual fragments exceeded twice the thickness of the laser fiber. Laser time was defined as the actual laser emission time obtained from the laser machine. Number of mucosal contacts represented the frequency of laser contacts with the mucosal surface. An independent observer assessed the number of contacts by analyzing changes in the laser-hitting sound (distinctive alterations in the sound occur when the laser makes contact with the mucosa of the kidney model) and by reviewing visual information from ureteroscopic images during the procedure. Total energy was collected from the laser device records and represented the cumulative energy of laser emission until stone-free status was achieved.

### Assessment and statistical analyses

Data analysis was performed using IBM SPSS version 26.0 Software (IBM, Armonk, NY, USA). Continuous variables were presented as the mean ± standard deviation. The differences between the three groups in continuous variables were analyzed using the one-way ANOVA test or the Kruskal–Wallis test depending on the data distribution. Post-hoc analysis was conducted using the Games–Howell test, Bonferroni correction, or Scheffe test, as appropriate based on the data characteristics. Differences between the two participants were analyzed using the *t* test and Mann–Whitney *U* test, depending on the normality of the data. A *P* value of less than 0.05 (two-sided) differences was considered statistically significant for all tests.

## Results

Tables 1, 2, and 3 present the outcomes for fragmentation time, lasing time, number of mucosal contacts, and total energy in the groups of M, RNR, and RR for all surgeons (Overall) and each individual surgeon. Video 1 demonstrates the laser lithotripsy procedure in each group (M, RNR, and RR).

**Table 1:** Fragmentation time, laser time, number of mucosa contacts, and total energy for each group, presented for overall (surgeon A and B) and each individual surgeon

		Fragmentation time (s)	Laser time (s)	No. of mucosa contacts	Total energy (Wh)
Overall	Group M	1698.2 ± 370.9	1049.9 ± 143.8	2.1 ± 2.3	3.9 ± 0.6
	Group RNR	1270.9 ± 269.2	894.9 ± 205.7	0.8 ± 1.7	3.4 ± 0.8
	Group RR	1103.7 ± 157.9	791.6 ± 148.7	0.7 ± 1.3	3.0 ± 0.6
	P-value	0.000	0.005	0.036	0.011
Surgeon A	Group M	1360.4 ± 69.9	957.8 ± 49.5	2.0 ± 1.0	3.5 ± 0.2
	Group RNR	1153.3 ± 304.4	839.9 ± 238.9	0.4 ± 0.8	3.2 ± 0.9
	Group RR	1059.3 ± 200.1	774.6 ± 179.3	0.3 ± 0.5	2.9 ± 0.7
	*P*-value	0.102	0.123	0.011	0.348
Surgeon B	Group M	2036.0 ± 139.4	1142.0 ± 151.2	2.2 ± 3.3	4.3 ± 0.6
	Group RNR	1388.4 ± 179.3	950.0 ± 166.0	1.1 ± 2.3	3.6 ± 0.6
	Group RR	1148.1 ± 96.8	808.7 ± 122.8	1.1 ± 1.8	3.1 ± 0.6
	*P*-value	0.000	0.005	0.633	0.016

Values are presented as the mean ± standard deviation

**Table 2:** P-values for intergroup comparisons derived from post-hoc analyses, presented for overall (surgeon A and B) and each individual surgeon

		Fragmentation time (s)	Laser time (s)	No. of mucosa contacts	Total energy (Wh)
Overall	Group M vs Group RNR	0.012	0.162	0.048	0.196
	Group M vs Group RR	0.001	0.003	0.093	0.011
	Group RNR vs Group RR	0.160	0.431	1.000	0.350
Surgeon A	Group M vs Group RNR	–	–	0.030	–
	Group M vs Group RR	–	–	0.018	–
	Group RNR vs Group RR	–	–	1.000	–
Surgeon B	Group M vs Group RNR	0.000	0.118	–	0.164
	Group M vs Group RR	0.000	0.005	–	0.016
	Group RNR vs Group RR	0.013	0.229	–	0.393

**Table 3:** Comparison of fragmentation time, laser time, number of mucosal contacts, and total energy between surgeons

	Fragmentation time (s)			Laser time (s)		
	Group M	Group RNR	Group RR	Group M	Group RNR	Group RR
Surgeon A	1360.4 ± 69.9	1153.3 ± 304.4	1059.3 ± 200.1	957.8 ± 49.5	839.9 ± 238.9	774.6 ± 179.3
Surgeon B	2036.0 ± 139.4	1388.4 ± 179.3	1148.1 ± 96.8	1142.0 ± 151.2	950.0 ± 166.0	808.7 ± 122.8
*P*-value	0.000	0.137	0.319	0.050	0.259	0.685

	No. of mucosa contacts			Total energy (Wh)		
	Group M	Group RNR	Group RR	Group M	Group RNR	Group RR
Surgeon A	2.0 ± 1.0	0.4 ± 0.8	0.3 ± 0.5	3.5 ± 0.2	3.2 ± 0.9	2.9 ± 0.7
Surgeon B	2.2 ± 3.3	1.1 ± 2.3	1.1 ± 1.8	4.3 ± 0.6	3.6 ± 0.6	3.1 ± 0.6
*P*-value	0.383	0.902	0.318	0.039	0.338	0.491

In the overall results of the two surgeons, fragmentation time was significantly reduced to 74.8% in group RNR (*P* = 0.012) and 65.0% in group RR (*P* = 0.001), compared to group M. Fragmentation time for the relatively more experienced surgeon (Surgeon A) remained comparable across all three groups. Fragmentation time for the relatively less experienced surgeon (Surgeon B) was significantly decreased in both RNR and RR groups compared to group M (*P* < 0.001 and *P* < 0.001, respectively). Furthermore, fragmentation time was significantly shorter in group RR compared to group RNR for surgeon B (*P* = 0.013).

In the overall results of the two surgeons, laser time was significantly shorter in group RR compared to group M (*P* = 0.003). The laser time for surgeon A was comparable across all three groups. The laser time for surgeon B demonstrated a significant decrease in group RR compared to group M (*P* = 0.005).

In the overall results of the two surgeons, number of mucosal contacts was significantly reduced to 37.4% in group RNR (*P* = 0.048), and it was 34.0% in group RR, compared to group M. For surgeon A, the number of mucosal contacts significantly decreased in both RR and RNR groups compared to group M (*P* = 0.030 and 0.018, respectively). The number of mucosal contacts for surgeon B was comparable across all three groups.

In the overall results of the two surgeons, total energy was significantly decreased in group RR compared to group M (*P* = 0.011). For surgeon A, the total energy was comparable across all three groups. Total energy for surgeon B showed a significant decrease in group RR compared to group M (*P* = 0.016).

In group M, surgeon A, with more RIRS experience, showed significantly reduced fragmentation time (*P* < 0.001), laser time (*P* = 0.050), and total energy (*P* = 0.039) compared to surgeon B, who had relatively less RIRS experience. However, fragmentation time, laser time, and total energy were comparable between the two surgeons in both the RNR and RR groups.

## Discussion

RIRS remains a challenging technique that relies on successful navigation through the entire ureter and renal calyces, efficient stone fragmentation, and complete removal of fragments, all within a dynamically moving organ [5]. The surgeon’s proficiency plays a crucial role in the success of RIRS procedures. It has been reported that urologists need to perform approximately 40 to 60 procedures to reach a minimal level of proficiency [10, 11]. Resources such as models, hands-on courses, and fellowship programs are available to train urologists in performing RIRS [8]. However, these training methods primarily focus on teaching the technical aspects of the procedure and do not adequately address the impact of respiratory-related renal movements. This aspect deserves greater attention, as the unpredictable mobility of stones can significantly affect the procedure’s efficacy. We believe adjusting for renal movements associated with respiration can substantially improve the outcomes of RIRS. To the best of our knowledge, this is the first investigation to demonstrate the feasibility of using a robotic platform for RIRS in the context of stone fragmentation and mucosal damage under respiratory motion.

The study demonstrated improved outcomes in the robotic-assisted procedure compared to the manual procedure, even in the absence of respiratory synchronization in the robotic procedure. With robotic assistance, the surgeons were able to efficiently and safely perform laser lithotripsy under the patient respiratory motion. A robot can stabilize the scope’s position during laser fragmentation, enabling operators to focus entirely on managing laser firing according to respiratory movements once the optimal scope and laser fiber positions are determined. In contrast, manual procedure requires the operator to rely on instinctive efforts to manage respiratory movements, along with additional challenges, such as efforts to maintain the scopes’ position, adjusting the operator’s posture, managing irregular breaks due to wrist/shoulder fatigue, and dealing with other unpredictable variables.

Respiratory synchronization could be easily achieved in the robotic procedure by simply rotating a scroll wheel via console handle, a task that is difficult to accomplish in manual procedures. The results suggested that robotic assistance with respiratory synchronization is particularly effective for a less experienced surgeon. In contrast, for a more experienced surgeon, the impact of robotic assistance appears less pronounced, as he is relatively more skilled at dealing with the respiratory motion during laser fragmentation. In addition, the results implied that the robotic-assisted RIRS could mitigate the difference in performance due to variations in the skill level of the surgeons. Given the limited number of participants, further research is required to comprehensively evaluate the effectiveness of respiratory synchronization based on the surgeon’s level of expertise in RIRS.

Several techniques have been proposed in the literature to minimize the kidney movement resulting from respiratory motion. In techniques such as fragmentation or pop-dusting, it is crucial to maintain a consistent and stable distance for the laser fiber from the stone and the mucosa. Temporarily pausing respiration during the lithotripsy can, therefore, be beneficial [12, 13]. Various anesthesia techniques, such as high-frequency and small-volume mechanical ventilation, have been proposed to address the stone movement within the operative field due to mechanical ventilation [14]. Other methods, including high-frequency ventilation [15], intraoperative apnea [16], abdominal compression [17], and general anesthesia with low ventilation [5], have also been investigated. Despite the efforts, existing approaches, such as artificially reducing respiration through mechanical ventilation or inducing hyperventilation or apnea, pose ongoing concerns regarding the safety and stability of surgical procedures, which may burden both patients and surgeons. It is essential to explore solutions that can significantly enhance surgical efficiency and safety by accounting for respiratory-induced kidney movement. Consequently, the authors propose the need for a system that enables long-term stable control of renal movement during lithotripsy, utilizing the consistent amplitude and cycle of respiration for an automated response. This would allow the robotic system to manage respiratory movement, while the surgeon focuses on active control of the procedure.

The exact quantification of kidney motion during respiration is challenging. The kidney experiences movement with deep respiration, with the superior pole reaching a maximum displacement of up to 39 mm during both inspiration and expiration, while the inferior pole can move as much as 43 mm [18]. During RIRS, the patient’s respiratory cycle can be consistently regulated through the anesthetic machine. However, the magnitude of respiratory excursion can vary significantly depending on the patient’s physiological characteristics. If the respiratory excursion is the same for cases, it may lead to a learning effect for the surgeon and introduce potential data errors. To minimize errors, we randomly assigned respiratory excursions of 10, 15, and 20 mm without informing the surgeon. This approach was intended to reduce learning biases and minimize potential errors as much as possible.

One significant factor contributing to the prolonged operation time is mucosa contact during surgery, leading to surgical bleeding. This can pose challenges, particularly for novice surgeons, as it impairs visibility and makes it difficult to identify the location of the stone. It prolongs surgical time and increases the risk of complications such as hematuria, pyelonephritis, or sepsis due to mucosal contraction caused by direct laser firing when visual clarity is compromised [19]. While Ho:YAG laser energy can be safely used if the tip of the laser fiber is positioned more than 2 mm away from the urothelium, challenges persists in the actual surgical environment. The kidneys and upper ureter constantly in motion due to the patient’s breathing, which can pose a risk during laser procedures. In addition, the production of stone dust during laser emission often leads to poor visibility, further increasing the potential for tissue damage. Close monitoring of the urinary system’s movement in sync with the patient’s respiratory pattern is crucial to mitigate these complications. Ensuring that the tip of the laser fiber remains safe throughout respiratory motion becomes essential to prevent adverse outcomes.

Surgeons in the field are often exposed to work-related musculoskeletal fatigue and radiation exposure risk. Prolonged standing, maintaining static postures, while wearing heavy lead gowns, repetitive foot pedal actions, strain on wrist and thumb muscles, and knee pain from holding the uretroscope can have all detrimental physical effects on surgeons. Although the current study did not specifically address these issues, robots can offer the advantage of stabilizing the ureteroscope throughout the procedure, regardless of its duration. This alleviates strain on the surgeon’s muscles and allows for more precise and delicate surgical maneuvers.

### Limitations

This study has several limitations. First, the experiment was conducted exclusively in the upper pole, rather than in various renal locations. This decision was made, because the upper pole is the location, where the effectiveness of respiratory synchronization, utilizing the back-and-forth movement of the laser fiber, would be maximized. This is due to the kidney’s predominant back-and-forth motion induced by respiration, which is clearly observed in the ureteroscopic view. In addition, during laser lithotripsy, it is common practice to position stones in a dependent location, such as the upper pole, to optimize fragmentation efficiency. Although further investigations across various renal locations are needed, the primary significance of this study lies in evaluating the feasibility of the robotic assistance in the most effective application while reflecting practical clinical scenarios. Second, this was an in vitro study conducted using a kidney simulator model, which limited the ability to account for factors, such as hematuria during actual surgeries. The effect of hematuria will be investigated in the further in-vivo study. Nevertheless, robotic assistance may help reduce mucosal contacts and potentially mitigate bleeding-related problems as well. Third, the sample size is limited and not determined through statistical calculations. This study was designed as a pilot investigation to explore the preliminary outcomes of robotic-assisted laser lithotripsy with respiratory synchronization. Consequently, the sample size was determined based on practical considerations, including available resources and the exploratory nature of the study, rather than through a formal sample size calculation. Based on the findings of this study, further study with larger, adequately powered sample sizes and a greater number of participants with varying levels of expertise will be conducted.

## Conclusion

Robotic-assisted RIRS resulted in reduced fragmentation time, laser time, mucosal contacts, and total energy compared to manual RIRS during laser lithotripsy. The incorporation of respiratory synchronization in robotic-assisted RIRS reduced laser time compared to manual RIRS and shortened the fragmentation time compared to the robotic-assisted RIRS without respiratory synchronization, particularly for a less experienced surgeon. These initial results demonstrated the feasibility of robotic-assisted RIRS with respiratory synchronization, highlighting its potential to improve procedural efficiency and safety. The findings underscore the importance of continued research in robotic-assisted RIRS and the need for comprehensive investigations to establish the broader applicability and safety implications of these procedures.

## Supplementary Information

**Additional file 1: Video 1.** Differences of stone fragmentation without and with respiratory synchronization.

## Data Availability

No datasets were generated or analysed during the current study.

## Abbreviations

M: Manual procedure
RNR: Robotic procedures without respiratory synchronization
RR: Robotic procedures with respiratory synchronization
RIRS: Retrograde intrarenal surgery
PCNL: Percutaneous nephrolithotomy
